# Integration of Metal–Organic Polyhedra onto a Nanophotonic Sensor for Real-Time Detection of Nitrogenous Organic Pollutants in Water

**DOI:** 10.1021/acsami.3c07213

**Published:** 2023-08-11

**Authors:** Olalla Calvo-Lozano, Laura Hernández-López, Leyre Gomez, Arnau Carné-Sánchez, Cornelia von Baeckmann, Laura M. Lechuga, Daniel Maspoch

**Affiliations:** † Catalan Institute of Nanoscience and Nanotechnology (ICN2), CSIC, and Barcelona Institute of Science and Technology, Campus UAB, 08193 Bellaterra, Barcelona, Spain; ‡ Departament de Química, Facultat de Ciències, Universitat Autònoma de Barcelona, 08193 Bellaterra, Spain; § ICREA, Pg. Lluís Companys 23, 08010 Barcelona, Spain; ⊥ Catalan Institute of Nanoscience and Nanotechnology (ICN2), CSIC, CIBER-BNN, and Barcelona Institute of Science and Technology, Campus UAB, 08193 Bellaterra, Barcelona, Spain

**Keywords:** metal−organic polyhedra, nanophotonic sensor, interferometer, benzotriazole, imidacloprid, environmental monitoring

## Abstract

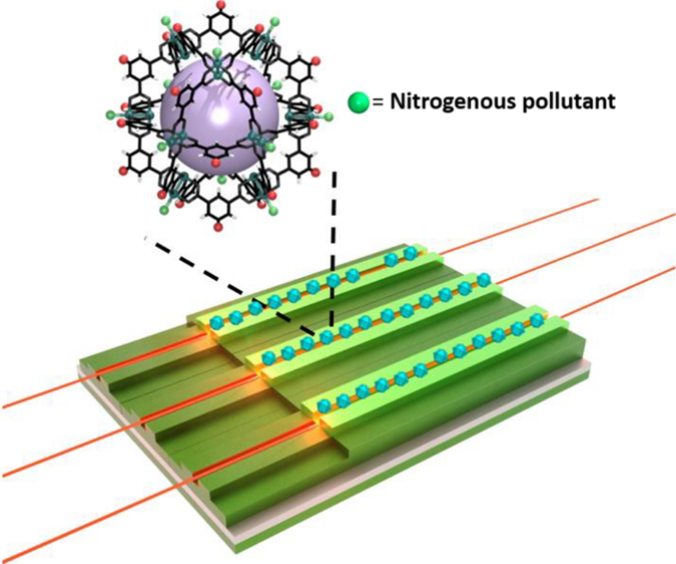

The grave health and environmental consequences of water pollution demand new tools, including new sensing technologies, for the immediate detection of contaminants in situ. Herein, we report the integration of metal–organic cages or polyhedra (MOCs/MOPs) within a nanophotonic sensor for the rapid, direct, and real-time detection of small (<500 Da) pollutant molecules in water. The sensor, a bimodal waveguide silicon interferometer incorporating Rh(II)-based MOPs as specific chemical receptors, does not require sample pretreatment and enables minimal expenditure of time and reagents. We validated our sensor for the detection of two common pollutants: the industrial corrosion inhibitor 1,2,3-benzotriazole (BTA) and the systemic insecticide imidacloprid (IMD). The sensor offers a fast time-to-result response (15 min), high sensitivity, and high accuracy. The limit of detection (LOD) in tap water for BTA is 0.068 μg/mL and for IMD, 0.107 μg/mL, both of which are below the corresponding toxicity thresholds defined by the European Chemicals Agency (ECHA). By combining innovative chemical molecular receptors such as MOPs with state-of-the-art photonic sensing technologies, our research opens the path to implement competitive sensor devices for in situ environmental monitoring.

## 1 Introduction

Ubiquitous pollution of natural water resources with anthropogenic contaminants such as agriculture products, fine chemicals, pharmaceuticals, and fuels has created a crisis for both human health and the environment.^1^ Although there are highly accurate and sensitive analytical techniques for environmental monitoring, they present major practical limitations: for instance, most chromatography techniques require expensive, specialized laboratory equipment, and arduous sample-preparation procedures. Accordingly, there is a pressing need for simpler tools for the rapid, accurate detection of water pollutants in situ.

A particularly promising field for environmental monitoring is sensing. For example, evanescent wave (EW) optical sensors—especially interferometric devices—have garnered attention for accurate, highly sensitive, real-time, and label-free detection of analytes. In these sensors, contact with the target analyte provokes an extremely subtle change in the refractive index (RI) on their surface, which in turn alters the properties of the propagated light (e.g., phase, intensity, etc.) through their EW. Thus, the extent to which the light-propagation changes can be correlated with the analyte concentration. Among interferometric sensors, the bimodal waveguide (BiMW) sensor has demonstrated its potential for biosensing of various analytes in diverse applications, including hormones, microRNAs or bacteria in clinical diagnosis,^2−4^ and algaecides or insecticides in environmental monitoring.^5,6^ Moreover, the BiMW sensor is based on silicon technology and microelectronic fabrication, thereby enabling its mass production with multiplexed configuration and facilitating its integration into portable sensing devices.

The development of biosensors such as those based on EW sensors generally requires the use of biomolecules (e.g., antibodies or DNA probes) as biorecognition elements or biological receptors.^7^ Due to the fragility of these receptors, their production and handling can be both costly and complex: for example, as concerns the working temperature, pH, and/or sterility.^8^ Another challenge for EW sensors is that the detection of analytes smaller than 500 Da is not trivial, especially those at low concentrations.^9^ Consequently, EW sensors often demand more arduous sensing strategies, such as indirect competitive immunoassays that require separate steps for bioconjugation, amplification, and/or preconcentration of the target analyte(s).^5,6^ A promising solution to address both of these shortcomings is the use of porous materials as alternative receptors in BiMW sensors. Although the practical application of extended and crystalline porous metal–organic frameworks (MOFs) has previously been demonstrated with BiMW sensors for gas sensing,^10^ these sensors are hindered by their inherent grain boundaries when the MOF is located/assembled on the sensing surface. Indeed, with MOFs, the film textures (e.g. grain/crystallites sizes, grain boundaries, and mesopores), crystalline orientations, and exposed crystal facets crucially induce light-scattering that suppresses the propagation of the EW and, consequently, directly affects the analytical performance of the optical sensor.^11^ This hurdle has very recently been circumvented by miniaturizing MOF (in particular, ZIF-8) particles to smaller than 30 nm and then assembling them in dense, transparent optical films.^10^

We hypothesized that one strategy to further miniaturize the molecular receptors to be incorporated in the nanophotonic sensors would be to use molecular metal–organic cages/polyhedra (MOCs/MOPs). MOPs offer rich covalent and coordination chemistry to enable stoichiometric functionalization of sensor surfaces^12^ to create devices that can recognize and bind specifically target analytes. Additionally, unlike MOFs or covalent organic frameworks (COFs), they can be dissolved and therefore easily processed.

The capability of some MOPs to trigger luminescent and catalytic processes in the presence of target analytes has been harnessed to develop colorimetry or fluorescence-based bulk sensors (i.e., unprocessed powders).^13−19^ However, given that incorporating MOPs onto the surface of analytical devices is difficult, it has only been reported twice in the literature. In the first example, Li and co-workers immobilized a Cu(II)-based lantern MOP onto the surface of a plasmonic substrate through coordinative interactions, creating a Raman sensor that exhibited good surface-enhanced Raman scattering enhancement, which they exploited to detect nitro-organic compounds in dichloromethane solutions in the micro- to nanomolar range.^20^ Later, Smet and co-workers similarly decorated the surface of a silicon nanowire-based field-effect transistor with Cu(II)-based lantern MOPs to afford a sensor for the detection of 2,4,6-trinitrotoluene in ethanol with a detection limit below the nanomolar level.^21^ Unfortunately, the use of hydrolytically unstable Cu(II)-based MOPs as receptors, and the reversible coordinative attachment methods, precludes the use of the aforementioned sensors for real-time monitoring of aqueous samples.

**Figure 1:**
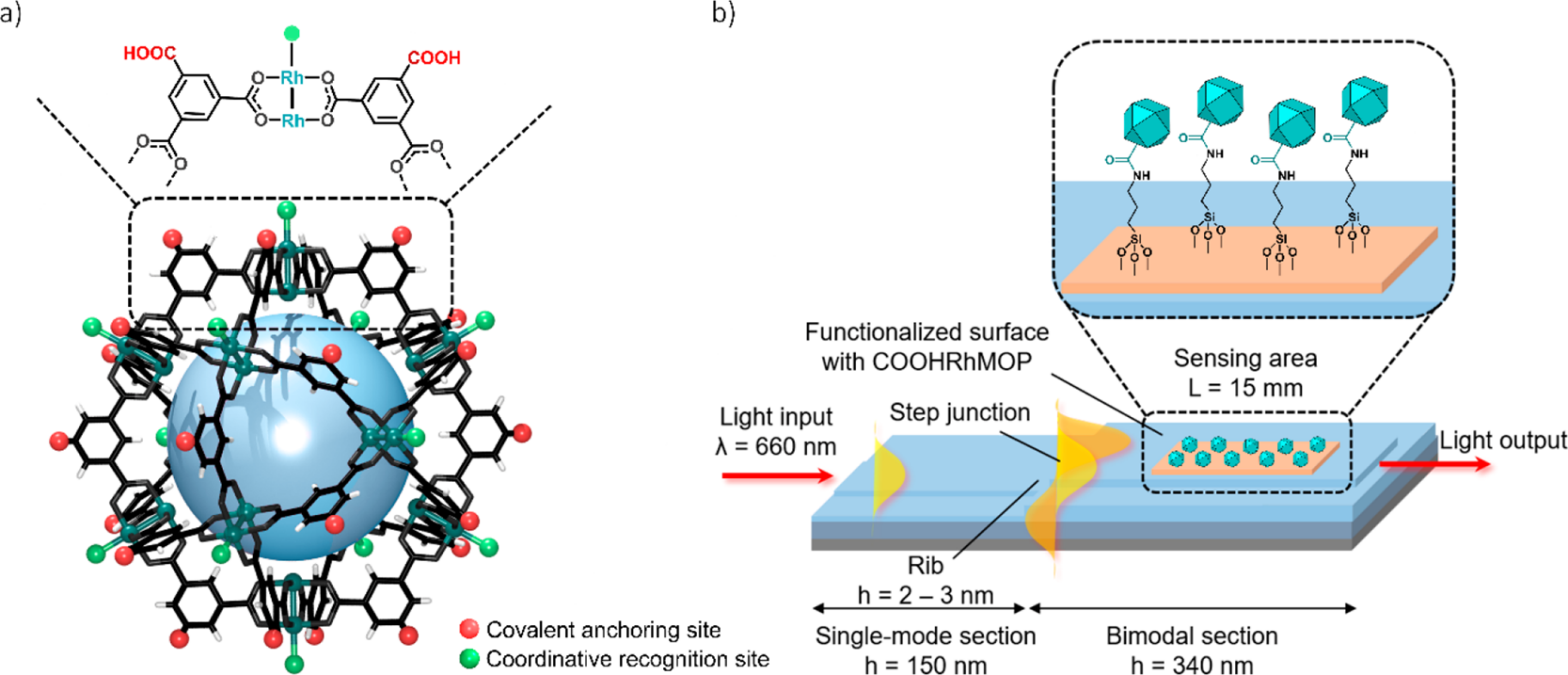
(a) Structure of cuboctahedral Rh(II)-MOP, highlighting the 5-positions in the organic backbone (covalent anchoring sites) and the axial sites of its dirhodium paddlewheels (coordinative recognition site). (b) Schematic of the MOP-BiMW sensor, showing its main characteristics, including the covalently attached Rh(II)-MOP.

In this study, we explored the implementation of robust, hydrolytically stable MOPs onto a BiMW sensor for environmental monitoring of aqueous samples contaminated with industrial and agrochemical pollutants. We selected the robust Rh(II)-based cuboctahedral [Rh_2_(COOH-bdc)_2_]_12_ MOP (hereafter named *COOHRhMOP*, where COOH-bdc is 5-carboxy-1,3-benzenedicarboxylate)^22^ as a receptor, as it can efficiently capture and remove nitrogenous pollutants from water,^23^ through the coordination reactivity of the 12 exposed axial sites of the Rh(II)-paddlewheels (Figure 1a, green dots). Additionally, this Rh-MOP is water-stable in a broad pH range (i.e., pH = 1–12)^23^ and is functionalized with a carboxylic acid group at the 5-position of the phenyl ring of each bdc linker, such that its external surface is functionalized with a total of 24 carboxylic acid groups (Figure 1, red dots). Therefore, these carboxylic groups can be used to immobilize the Rh-MOPs onto the previously amine-functionalized BiMW sensor surfaces through amide couplings. Using this surface chemistry, we were able to integrate COOHRhMOPs onto the BiMW surface, obtaining a sensor for rapid (<15 min) detection of low-weight coordinating analytes in water, without the need for any sample pre-treatment (Figure 1b). We validated our optical MOP-BiMW sensor for the detection of two harmful aqueous pollutants: the widely used industrial corrosion inhibitor 1,2,3-benzotriazole (BTA, MW = 119.12 g/mol) and the systemic insecticide imidacloprid (IMD, MW = 255.661 g/mol), both of which have severe effects on animal and human health.

## 2 Results and Discussion

### 2.1 Synthesis and Recognition Capabilities of the MOP-Based Sensor Surface

To fabricate the MOP-BiMW sensor, we began with the integration of MOPs, as the selective receptor layer, onto the Si_3_N_4_ waveguide surface of the BiMW (Figure 1b). To this end, the surface sensor was initially silanized with aminopropyltrimethoxysilane (APTES) to functionalize it with amino groups, which enabled subsequent immobilization of COOHRhMOP via carbodiimine-based amide coupling. Although this procedure is employed for silicon surface functionalization with various biomolecules,^24^ to the best of our knowledge, it has never previously been applied to MOPs. The solution-phase coupling was evaluated by reacting COOHRhMOP with a model aliphatic amine molecule, propylamine, in 2-(*N*-morpholino)ethanesulfonic acid (MES)-buffered solution (pH = 6) in the presence of the coupling agents 1-ethyl-3-(-3-dimethylaminopropyl) carbodiimide (EDC, 0.2 M) and *N*-hydroxysuccinimide (NHS, 0.05 M) at room temperature for 12 h. This model reaction afforded the amide-coupled-RhMOP in a yield of 50% (i.e., 12 of the 24 COOH groups were coupled with the model amine molecule), thus demonstrating the viability of the coupling chemistry for immobilization of COOHRhMOP onto the APTES-modified sensor surface (Figures S2–S5). Thus, the APTES-functionalized sensor was incubated in a COOH-RhMOP aqueous solution under the same reaction conditions as described above. After the amide coupling, the surface of the sensor chip was thoroughly washed with water to remove any non-covalently attached MOP. The presence of Rh(II) on the sensor chip was confirmed by in situ X-ray photoelectron spectroscopy (XPS) and upon analysis of acid-digested samples by inductively coupled plasma-mass spectrometry (ICP-MS) (Figure S7 and Table S1).

**Figure 2:**
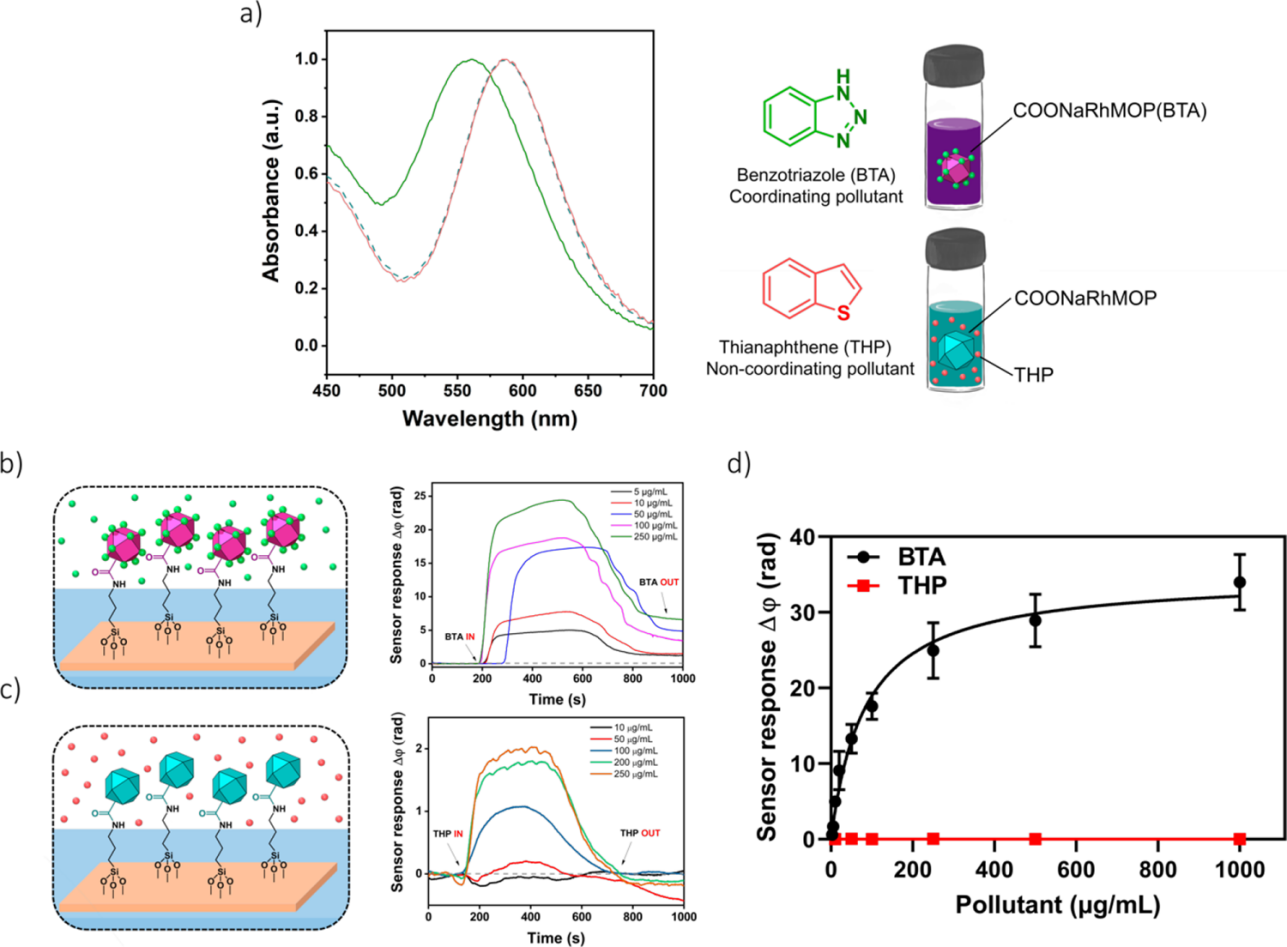
(a) Left: UV–vis spectra of COONaRhMOP in water before (blue dashed line) and after the addition of either BTA (green line) or THP (pink line). Right: schematic of the resulting solution after adding BTA or THP to an aqueous solution of COONaRhMOP and the molecular structure of these pollutants. (b) From left to right: schematic of the response mechanism for the immobilized COOHRhMOP on the BiMW sensor surface in the presence of BTA and real-time sensorgram of different concentrations of BTA with the COOHRhMOP/BiMW sensor in Milli-Q water. (c) From left to right: schematic of the absence of response of the immobilized COOHRhMOP on the BiMW sensor surface in the presence of THP and real-time sensorgrams of different concentrations of THP with the COOHRhMOP/BiMW sensor in Milli-Q water. (d) Calibration curves for BTA and THP. In both calibration curves, each signal corresponds to the mean ± SD of triplicate measurements on different batches of BiMW sensor chips.

Having successfully immobilized the COOHRhMOP onto the sensor surface, we next assessed the performance of the resultant MOP-BiMW sensor for the detection and quantification of organic pollutants in water. To this end, we exploited the reactivity of the axial sites of the Rh(II)-paddlewheels units, which exhibit high affinity toward Lewis bases such as N-donor ligands. These interactions can be detected in solution by monitoring the spectroscopic changes in the bands centered in the range from 500 to 600 nm (λ_max_), which corresponds to the π* → σ* transitions of Rh-Rh bonds (Figure 2a).^25^ We envisaged that this coordination chemistry could be transferred to the BiMW sensor surface such that the coordinative pollutant-MOP interaction could be transduced into an optical analytical signal.

We first evaluated our MOP-BiMW sensor for the detection of BTA (Figure 2b). This began with comparing its response to that of bare or APTES functionalized BiMW sensors (i.e., two lacking COOHRhMOP). Thus, a solution of BTA in Milli-Q water (concentration: 1000 μg/mL) was sequentially injected through the three sensors (Figure S8). Interestingly, no sensor responses (phase changes) were observed in the bare BiMW sensor, thus ruling out any nonspecific adsorptions of the analyte onto the inert silicon nitride or amine-functionalized sensor surface (Figure S8). Contrariwise, and importantly, a significant phase variation signal was observed for the MOP-BiMW sensor (Figure S8). Next, the calibration and limit of detection (LOD) of the MOP-BiMW sensor were calculated according to an additive-calibration curve (concentrations: 5–1000 μg/mL; Figures 2b,d and S9), which revealed an LOD of 0.064 μg/mL, an excellent value that is several orders of magnitude below the EC_50_ (i.e., the concentration that is lethal to 50% of the population of a species) for aquatic organisms (940 μg/mL), as established by the European Chemicals Agency (ECHA).^26^ Furthermore, this LOD for BTA is also well below the no observed-effect concentration (NOEC, the highest concentration proven to produce no adverse effects on aquatic organisms) of 1 μg/mL.

Since we had hypothesized that the recognition mechanism—and consequently, the sensing process—is derived from the coordinative capabilities of the Rh(II) axial coordination sites, we next sought to confirm our hypothesis by testing the sensor against a noncoordinating analyte. To this end, thianaphthene (THP; MW = 134.20 g/mol), a molecule with similar shape, size, and polarity to BTA, was chosen as a representative non-coordinative analyte. Although Rh(II) ions also show an excellent affinity for S-donor molecules, the aromaticity of THP precludes the interaction with the Rh(II) axial sites, as the free electron pair at the S atom is delocalized within the π ring system (Figure 2a). As expected, at all tested concentrations of THP, the sensor exhibited a negligible response, thus confirming that its previously observed response toward BTA did indeed proceed by coordinative recognition (Figure 2c). By extension, these results confirmed that the selective COOHRhMOP-pollutant coordinative interactions transfer to the sensor surface.

**Figure 3:**
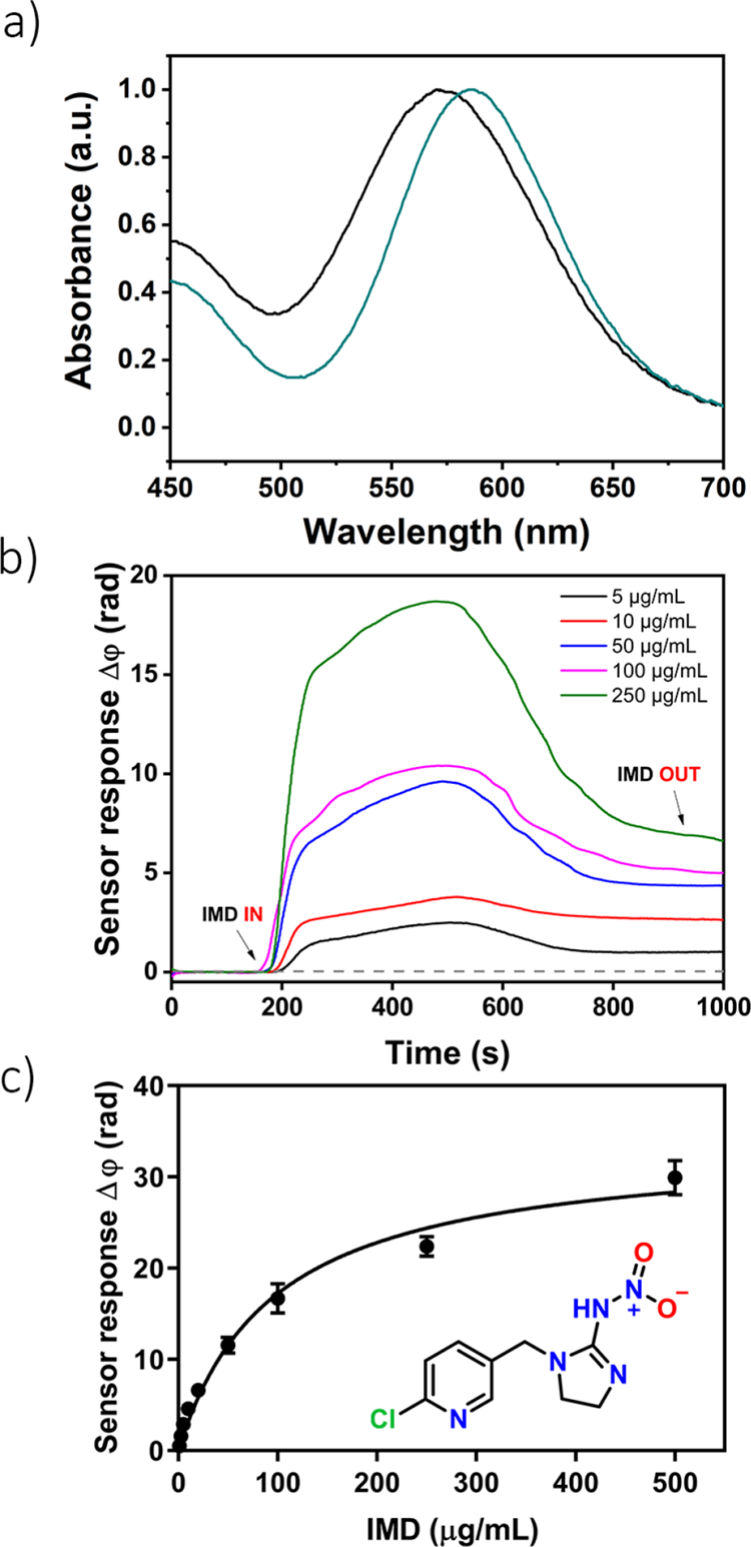
(a) UV–vis spectra of COONaRhMOP in water before (blue line) and after (black line) the addition of IMD. (b) Real-time sensorgram of different concentrations of IMD in the presence of the COOHRhMOP/BiMW sensor in Milli-Q water. (c) Calibration curve and molecular structure of IMD. Each signal corresponds to the mean ± SD of triplicate measurements on different batches of BiMW sensor chips.

Next, we tested our MOP-BiMW sensor for the detection of IMD in water (Figure 3a). Analogously to the case of BTA, the sequential addition of increasingly concentrated solutions of IMD in Milli-Q water (range: 1–500 μg/mL) to the MOP-BiMW sensor induced a clear interferometric phase-shift, from which a calibration curve and an LOD (0.234 μg/mL) were calculated (Figures 3b,c and S10). The poorer LOD calculated for IMD relative to that for BTA was attributed to its lower coordinative affinity toward COOHRhMOP (Figure S6). Nevertheless, this LOD for IMD is still well below its EC_50_ for aquatic organisms, whose values range from 8.7 to 180 μg/mL.^27^

Overall, the results discussed above confirmed that the main interaction between the MOP-BiMW sensor and organic pollutants in water proceeds through the coordination of the pollutant to the exposed Rh(II) axial sites of the sensor surface-anchored COOHRhMOPs. We pondered whether the highly sensitive analytical response achieved by the MOP-BiMW sensor could be achieved without structuring Rh(II) paddlewheel clusters in a caged structure. To answer this question, we functionalized the sensing area of the BiMW with a discrete Rh(II)-based paddlewheel cluster with formula Rh_2_(bdc)_4_ (Figure S11).^28^ This metal–organic complex replicates a metal–organic fragment present in the MOP structure and presents four available, un-coordinated COOH groups, which makes it an ideal model compound to assess the impact of structuring Rh(II) sites on the analytical response of BiMW sensors. The sensitivity of the Rh_2_(bdc)_4_-BiMW sensor toward BTA was significantly lower than that obtained for the MOP-BiMW sensor under otherwise identical conditions (Figure S12). Specifically, the LOD obtained for the Rh_2_(bdc)_4_-BiMW sensor was 0.352 μg/mL. We attributed the superior performance of the MOP-BiMW sensor to its higher density of analytically active Rh(II) sites (i.e., 0.15 Rh(II) sites per nm^2^), which translates into a higher number of receptor sites on the sensor surface.

### 2.2 Reproducibility, Accuracy, and Robustness of the MOP-Based Sensors

To ascertain the reproducibility of the sensor, we next measured the inter-assay variability according to the results of replicate experiments run within different sensor chips, which we calculated as the coefficient of variation (CV; expressed as %). Encouragingly, the CV that we obtained for both BTA (8%) and IMD (9%) were below the recommended maximum variability (15%) for the performance of commercial sensors^29^ (Table 1). These results confirmed the good reproducibility and robustness of the sensing strategy where a very low variability may come from sensor chip functionalization with COOHRhMOP and/or sample handling and preparation.

**Table 1:** Sensor Reproducibility Study^a^

BTA (μg/mL)	mean ± SD (Δφ)	CV (%)	IMD (μg/mL)	mean ± SD (Δφ)	CV (%)
5	1.69 ± 0.14	8	5	2.87 ± 0.25	9
10	4.99 ± 0.46	9	10	4.58 ± 0.61	13
50	13.29 ± 1.54	12	50	11.54 ± 0.71	6
100	17.58 ± 1.42	8	100	16.69 ± 1.30	8
tap water					
5	5.38 ± 0.40	7	5	3.13 ± 0.16	5
10	9.09 ± 0.75	8	10	4.39 ± 0.30	7
50	18.76 ± 0.60	3	50	8.60 ± 0.25	3
100	24.88 ± 1.75	7	100	12.56 ± 1.27	10

a Interassay variability for BTA and IMD in Milli-Q and tap water. Evaluations in triplicate.

**Figure 4:**
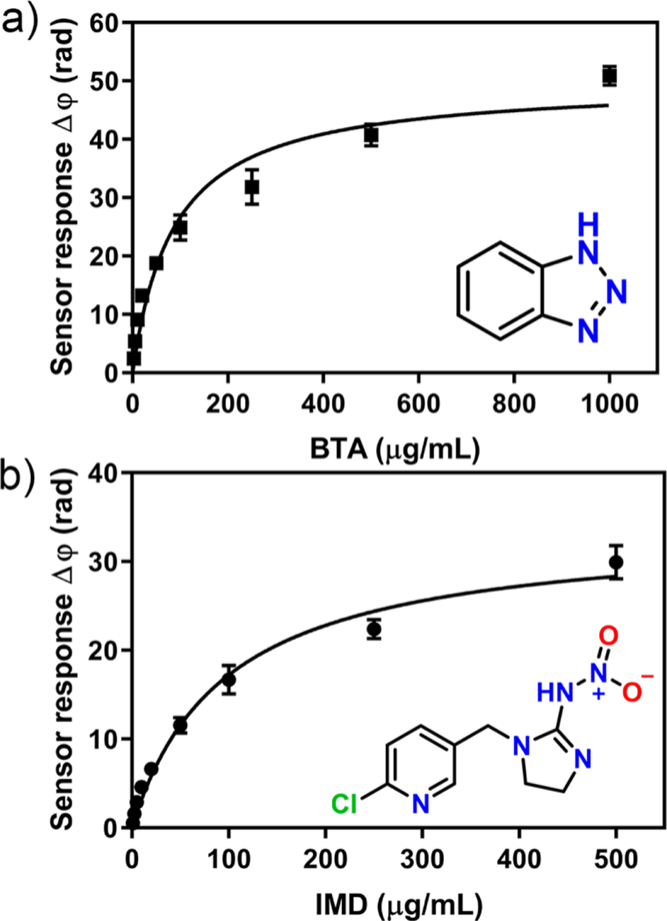
Calibration curves for the detection of BTA (a) and IMD (b) in spiked samples of tap water by the MOP-BiMW sensor. Each signal corresponds to the mean ± SD of triplicate measurements on different batches of BiMW sensor chips.

To test the performance of our MOP-BiMW sensor in a real-world application, we next screened it against samples of tap-water spiked with either BTA or IMD.^30,31^Figure 4 shows the calibration curves obtained for BTA and IMD. In both cases, the sensitivity remained in the low μg/mL range compared to the corresponding evaluations in Milli-Q water: 0.068 μg/mL for BTA and 0.107 μg/mL for IMD (Figure S13). The sensitivity was not affected by the presence of ions or other impurities in tap water, thus corroborating the potential of our sensor for in situ environmental control devices. Furthermore, although receptor saturation could be assumed above 500 μg/mL for BTA and 250 μg/mL for IMD, the MOP-BiMW sensor reported a wide dynamic range for pollutant concentrations from 1 to 250 or 100 μg/mL, respectively.

Next, we studied the reproducibility of the sensor using spiked tap-water samples (Table 1), again observing excellent sensor performance. These results further corroborated the suitability of our sensor for environmental monitoring of coordination analytes according to the previously mentioned FDA guideline for chemical sensors (max CV < 15%).

Finally, we decided to assess the accuracy of the sensor for the quantification of low concentrations of BTA or IMD within the dynamic range in spiked samples of tap water. As explained in detail in the Supporting Information, the accuracy was calculated considering the real and interpolated concentrations in each calibration curve after sample injections and sensor responses. In both cases, a good correlation was found between the actual concentrations and those obtained with the BiMW sensor: all accuracy values were within (or close to) the accepted accuracy range of 80–120% (Table 2). These values make our MOP-based optical sensor competitive against current analytical techniques for the detection of BTA and IMD (Table S2).

**Table 2:** Accuracy of the MOP-BiMW Sensor for Detection of BTA or IMD in Spiked Tap-Water Samples

sample #	[BTA], real (μg/mL)	[BTA], estimated (μg/mL)	accuracy (%)	sample #	[IMD], real (μg/mL)	[IMD], estimated (μg/mL)	accuracy (%)
S1	3.0	3.5	117	S6	3.0	3.5	118
S2	7.5	9.1	122	S7	7.5	9.4	125
S3	15.0	18.1	121	S8	15.0	17.2	115
S4	30.0	29.5	98	S9	30.0	33.1	110
S5	70.0	59.9	86	S10	70.0	63.7	91

For the detection of BTA, our sensor is just as efficient as that of other analytical techniques and tools such as surface-enhanced Raman scattering (SERS), differential pulse voltammetry (DPV), and high-performance liquid chromatography (HPLC), yet is faster because it does not require sample pre-treatment.^32−35^ However, for the detection of IMD, although its sensitivity is sufficient for environmental monitoring, it is far less sensitive than solid-phase extraction (SPE), which has a reported LOD of 0.0005 μg/mL.^36^ Nevertheless, our sensor exhibits a sensitivity close to or even better than that of other systems.^37,38^

## 3 Conclusions

In summary, we have combined the molecular recognition of discrete molecular cages with the sensing performance of optical sensors to create an Rh-MOP-functionalized BiMW sensor for rapid, direct, and real-time detection of water pollutants. This sensor can detect the common pollutants BTA and IMD in water in less than 15 min, exhibiting LODs as low as 0.068 μg/mL for BTA and 0.107 μg/mL for IMD. Importantly, we have demonstrated that the coordination chemistry of Rh-MOPs observed in solution can be transferred to the sensing surface of a BiMW device, as reflected in the fact that the sensor responded to these coordinating pollutants but did not respond to a noncoordinating analyte of similar size, shape, and polarity (thianaphthene, THP). We assessed the sensors for sensitivity, specificity, reproducibility, and accuracy. Significantly, according to ECHA guidelines for these contaminants, our technology exceeds the required analytical sensitivity (low μg/mL range) for both water contaminants, whose toxicity thresholds for aquatic life range from 5 to 900 μg/mL. Furthermore, all the analytical values obtained fall within the FDA guidelines for chemical sensors. We are confident that the integration of the specific reactivity and recognition capabilities of molecular cages—which could include coordination, electrostatic, and/or host guest-guest chemistry—into BiMW sensor technology will inform the design of future sensors for rapid, selective, and sensitive in situ environmental monitoring.

## Supporting Information Available

Detailed experimental procedures, materials and sensor characterization, and data analysis (PDF)
