# Supplementary material for: Integration of Metal–Organic Polyhedra onto a Nanophotonic Sensor for Real-Time Detection of Nitrogenous Organic Pollutants in Water
Source: ACS Appl Mater Interfaces. 2023 Aug 11;15(33):39523–9. doi: 10.1021/acsami.3c07213 (PMC10450679; doi:10.1021/acsami.3c07213)
Supplement: Supplementary file 1 — am3c07213_si_001.pdf [file am3c07213_si_001.pdf]

Supporting information

Integration of metal-organic polyhedra onto a nanophotonic sensor for real-time detection of nitrogenous organic pollutants in water

Olalla Calvo-Lozano,[a,§] Laura Hernández-López,[a,b,§] Leyre Gomez,[a,§] Arnau Carné-Sánchez,[b,a,*] Cornelia von Baeckmann,[a,b] Laura M. Lechuga[a,*] and Daniel Maspoch[a,b,c*]

[a] Catalan Institute of Nanoscience and Nanotechnology (ICN2), CSIC, and Barcelona Institute of Science and Technology, Campus UAB, 08193 Bellaterra, Barcelona, Spain. Email: arnau.carne@icn2.cat;laura.lechuga@icn2.cat; daniel.maspoch@icn2.cat

[b] Departament de Química, Facultat de Ciències, Universitat Autònoma de Barcelona, 08193 Bellaterra, Spain.

[c] ICREA, Pg. Lluís Companys 23, 08010 Barcelona, Spain.

Table of contents

S1. Materials and experimental methods

S1.1 Materials and characterization techniques

S1.2 Synthesis of COOHRhMOP and $Rh_2(BDC)_4$ cluster

S1.3 Fabrication and functionalization of the BiMW sensor

S1.4 Coordination capabilities of COOHRhMOP in aqueous solutions

S2. Characterization

S2.1 Model amide coupling between COOHRhMOP and propylamine

S2.2 Coordination capabilities of COOHRhMOP in aqueous solutions

S2.3 BiMW sensor surface characterization

S2.4 Characterization of the analytical performance of the Rh(II)-functionalized BiMW sensor surface.

S3. Data analysis

S4. References

S1. Materials and experimental methods

S1.1 Materials and characterization techniques

Rhodium (II) acetate, tetrabutylammonium fluoride (1 M in tetrahydrofuran - THF), isophthaloyl chloride, isophthalic acid (bdc), 2-(trimethylsilyl)ethanol, anhydrous pyridine, N,N-dimethylformamide (DMA), (3-aminopropyl)triethoxysilane (APTES), N,N-diisopropylethylamine (DIPEA), ninhydrin, N-(3-Dimethylaminopropyl)-N′-ethylcarbodiimide hydrochloride (EDC), N-hydroxysuccinimide (NHS), imidacloprid (IMD), thianaphtene (THP), toluene anhydrous, 2-(N-morpholino)ethanesulfonic acid (MES), and phosphate buffer saline tablets (PBS) were purchased from Sigma-Aldrich (US). 1,2,3-benzotriazole (BTA) was purchased from TCI chemical (Japan). Organic solvents (acetone, methanol and ethanol), hydrochloric acid (HCl, 37%), and nitric acid (HNO_3, 65%) were provided by Panreac (Spain). All reactants were used as provided with no further purification. The buffers employed were the following: MES buffer (0.1 M, pH 6), PBS (10 mM phosphate buffer, 2.7 mM KCl and 137 mM NaCl, pH 7.4). Milli-Q water was used for all solutions preparation except when tap water is specifically mentioned.

Ultraviolet-visible (UV−Vis) spectra were measured using an Thermo Scientific NanoDrop 2000 at room temperature (*ca.* 25 °C).

X-ray photoelectron spectroscopy (XPS) measurements were performed at room temperature with a SPECS PHOIBOS 150 hemispherical analyzer (SPECS GmbH, Berlin, Germany)) in a base pressure of $5x10^{-10}$ mbar using monochromatic Al-Kα radiation (1486.74 eV) as excitation source operated at 300 W. The energy resolution was measured by the FWHM of the Ag $3d_{5/2}$ peak for a sputtered silver foil was 0.62 eV. The spectra were calibrated with respect to the C1s at 284.8 eV.

Inductively coupled plasma mass spectrometry (ICP-MS) analyses were performed in an external company, Leitat, in an ICP-MS Agilent 7500. Quantification of Rh on each analyzed sensor was acquired after digestion of the samples in an analytical microwave (Anton Paar Multiwave 7000, Austria) at 280 ºC using 3 mL of HNO_3 (70%), 2 mL of HF (49%) and 1 mL of HCl (37%).

Matrix-assisted laser desorption/ionization-time of flight (MALDI-TOF) mass spectrometry (MS) measurements were performed using a 4800 Plus MALDI TOF/TOF (ABSCIEX – 2010) operating in positive-ionization mode using trans-2-[3-(4-tert-butylphenyl)-2-methyl-2-propenylidene]malononitrile (DCTB) as ionization matrix.

Nuclear magnetic resonance (NMR). All ^{1}H NMR spectra were recorded using a Bruker Avance NEO 300 NMR spectrometer at 25 °C. Chemical shifts (δ) are reported in ppm.

S1.2 Synthesis of COOH-RhMOP and $Rh_2(BDC)_4$ cluster

COOHRhMOP was synthesized following the protocol reported previously by us.[1] Briefly, using a three-step route, we synthesized a mono-protected 5-((2-(trimethylsilyl)ethoxy)carbonyl)isophthalic acid. This was followed by a complexation reaction adding Rh acetate in DMA under solvothermal conditions (100 ºC, 48 hours), obtaining a green solid (*i.e.*, the protected MOP) by water precipitation. The last step consisted of reacting the protected MOP with tetrabutylammonium fluoride solution 1 M in THF for 6 hours. The purity of COOHRhMOP was assessed by means of ^{1}H-NMR, MALDI-TOF and UV-Vis spectroscopy (**Figure S1**).

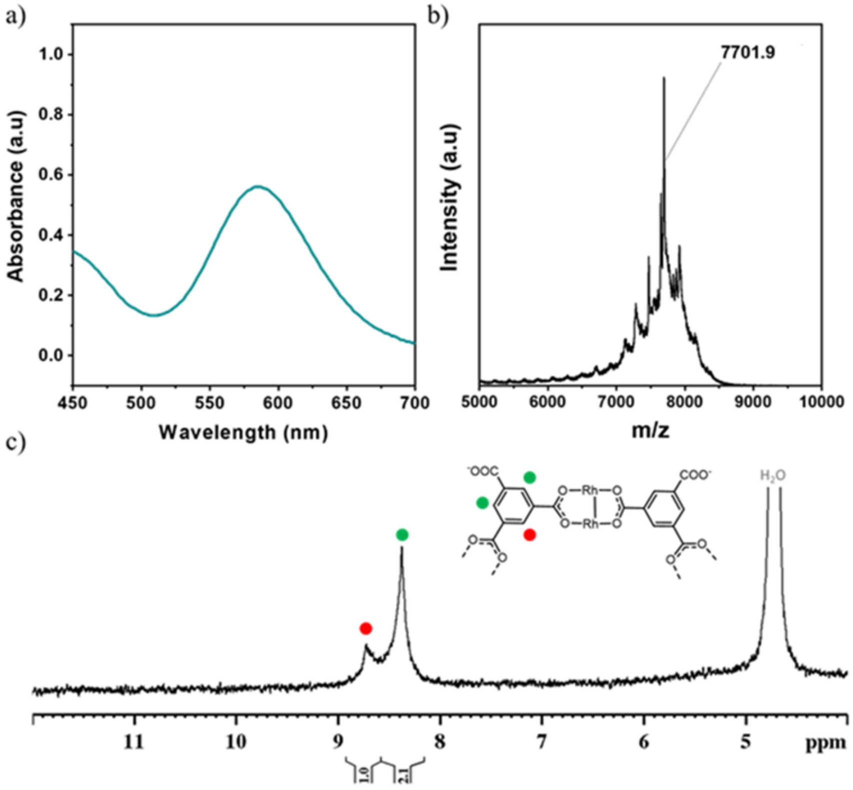

Figure S1. (a) UV-Vis absorbance data of COONaRhMOP (2 mg/mL) in aqueous solution. The presence of a λ_{max} centered at 885 nm confirms the integrity of the Rh(II)-Rh(II) paddlewheel. (b) MALDI-TOF spectra of COONaRhMOP. The weight corresponding to the formula $[Rh_{24}(COONabdc)_{24} - 12\ Na+ + 11\ H+]\cdot 3H_2O$ has been highlighted: expected = 7698; found = 7702. (c) ^{1}H NMR spectra in basic D_2O (pD = 10) of COONaRhMOP indicating the relative integrals of the assigned proton signals.

$Rh(BDC)_4$ cluster was synthesized following the reported protocol recently published by us.[2] The procedure entailed the synthesis of a half protected benzendicarboxylate ligand (1/2TMS-BDC) using 2-(trimethylsilyl)ethanol as the protecting group. Next, rhodium acetate (1 eq.; 0.15 mmol, 66.3 mg) and ½TMS-bdc (4 eq.; 0.6 mmol, 138.2 mg) were suspended in 20 mL of chlorobenzene. This mixture was stirred under reflux overnight. Then, the solvent was evaporated, and the crude product was redissolved in dichloromethane (40 mL). Reduced rhodium (0) was removed by centrifugation, and the green solution was again dried in vacuo. Purification by flash chromatography (silica gel, 2-10% EtOAc in DCM) yielded the desired product (43 % yield).

Amide coupling of COOHRhMOP with propylamine under homogenous conditions was performed as follows. 10 mg of COOHRhMOP were first solubilized in 2 mL MES-buffer (0.1 M, pH = 6) obtaining a green solution. Then, 48 eq of NHS in MES buffer (0.05 M) were added to the green solution upon stirring at room temperature. Afterwards, we added 48 molar equivalents of EDC (0.2 M in MES buffer) upon which the color of the solution changed to purple. After stirring for 30 minutes, 48 eq of propylamine were added and the reaction was left stirring overnight at room temperature. After 12 hours, the reaction mixture was centrifuged, and the resulting solid was washed three times with water (2 mL each time). Additionally, this solid was washed with acetone/ether and THF/ether mixtures to remove the remaining water and dried at room temperature for further characterization.

S1.3 Fabrication and functionalization of the BiMW sensor

BiMW sensor chip (1 x 3 cm; **Figure S1**) was fabricated at a wafer-scale using silicon photonic technologies at the ICTS/cleanroom from the IMB-CNM (CSIC).[3] Each chip integrates an array of 20 independent bimodal waveguides and 12 reference waveguides. Its working principle relies on the behavior of two transverse electric polarized light modes propagated through a waveguide. Briefly, light from a polarized diode laser (λ = 660 nm, HL6545MG, Thorlabs) is first confined through the waveguide core (rib thickness = 150 nm, height = 2 nm) in a single mode (fundamental). After a specific distance, the fundamental mode is coupled into a bimodal section through a step junction that allows the first propagating mode to emerge. These two modes travel across the sensing area (window in the bimodal section where detection happens) and exit the waveguide. Any refractive index changes in the sensing area (*e.g.* binding or detachment of molecules) disturbs the propagating modes through their evanescent fields and results in an interferometric phase shift (Δφ) between the fundamental and the first mode, modifying the light intensity distribution at the outlet. This intensity is recorded by a two-sectional photodetector (S4349, Hamamatsu) and processed with an acquisition card (NI USB-6361, National Instruments). To quantify the phase shifts between both modes, the interference signal is transformed into a linear one applying a previously developed all-optical phase modulation method based on Fourier Series deconvolution.[4] The BiMW sensor chip is placed on a Peltier thermoelectric cooler and a temperature controller (stabilization with an accuracy of 0.01 ºC) to compensate the intrinsic sensitivity of the BiMW interferometer to temperature fluctuations. To work with a continuous liquid flow, a fluidic system is incorporated into the system which includes: a pump (Gilson); a 6-port injection valve (Cheminert) that allows the injection of the different samples by sequential loading of the loop (160 µL); and a five-channel PDMS (polydimethylsiloxane) microfluidic cell, which seals the sensor chip (each liquid channel is 1.25 mm wide x 500 µm high).

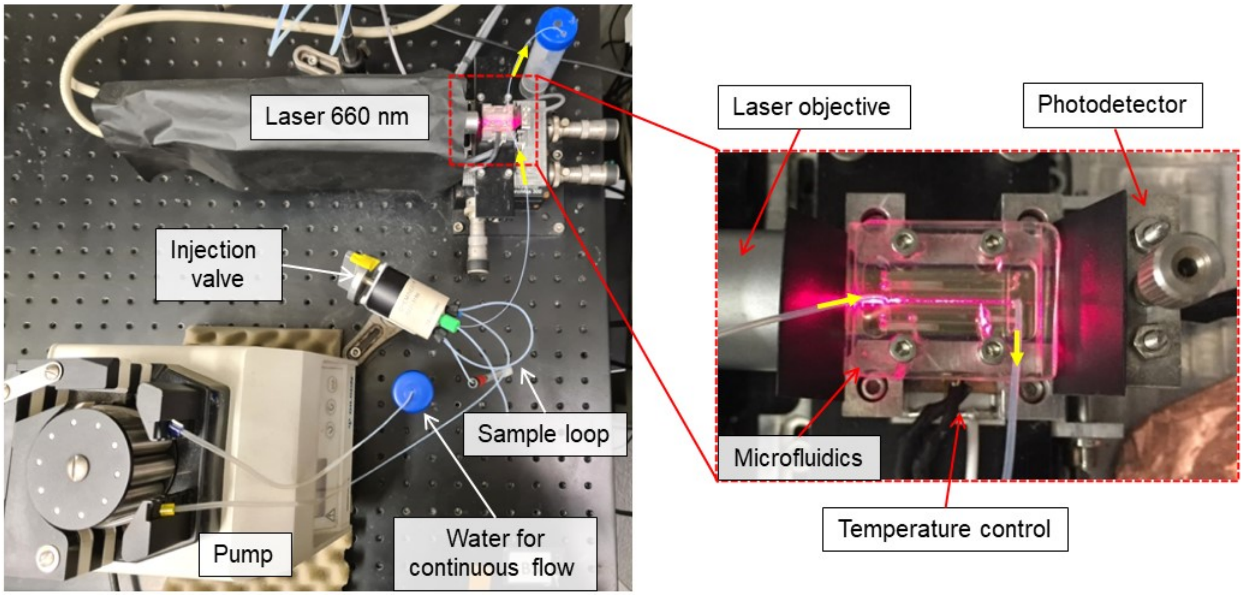

Figure S2. Photograph of a BiMW sensor chip placed on a holder with a temperature controller and with a polarized laser light (λ = 660 nm) coupled into a waveguide. The schematic below is a zoom of the bimodal waveguide showing its main characteristics.

COOH- and cluster- functionalized BiMW sensors were prepared by initially cleaning the BiMW sensor chip by 5 min sonication at 65 ºC in different solvents (sequentially, acetone, ethanol, and Milli-Q water) and 10 min in methanol/HCl 1:1 (v/v). Then, the sensor chip was rinsed with water and dried with a N_2 flow. Next, hydroxyl groups were generated on the sensor chip surface by placing it under oxygen plasma (Electronic Diener, Germany) for 5 minutes at 45 sccm gas flow, followed by immersion in a 15 vol.% HNO_3 solution at 95 ºC for 25 minutes. After rinsing with water and N_2 drying, the sensor chip was immediately functionalized with APTES, following the protocol previously detailed.[5] Briefly, the sensor chip was immersed in an anhydrous toluene solution with 1.0 vol.% APTES and 0.3 vol.% DIPEA for 1 hour at room temperature and N_2 atmosphere. After the silanization treatment, the sensor chip was rinsed with ethanol, dried with a N_2 stream and it was subjected to a curing process, by placing it in an oven at 110 ºC for 1 hour. The functionalized sensor chip was incubated with a solution containing 0.2 M EDC, 0.05 M NHS and 0.1 mg/mL COOHRhMOP or 0.1 mg/mL of the $Rh(BDC)_4$ cluster (dissolved in PBS) in MES buffer. The covalent immobilization takes place overnight at room temperature by reaction of the $-NH_2$ groups in the sensor chip surface with the –COOH groups of the COOHRhMOP or the

Rh(BDC)$_4$ cluster, creating an amide bond. After 12 hours, the sensor chip was rinsed generously with water and dried using N_2.

BTA and IMD were directly detected once the COOH-RhMOP was covalently attached to the sensor surface and the BiMW sensor chip was placed on the sensor platform. A set of fresh dilutions of the different pollutants (from 1 to 500 µg/mL for IMD and 1000 µg/mL BTA) was prepared in the corresponding working water (Milli-Q or tap). Having a flow of Milli-Q or tap water at a constant flow rate (20 µL/min), an additive assay was performed to obtain a calibration curve by injecting successive dilutions of increasing concentrations of BTA or IMD onto the sensor surface without further sample treatment.

S1.4 Coordination capabilities of COOHRhMOP in aqueous solutions.

The coordination capabilities of BTA, IMD and THP to the Rh(II) axial sites of COONaRhMOP were tested by using the following procedure. An aqueous solution of COONaRhMOP was prepared by adding 10 mg of COOHRhMOP (1.36 µmol) to 5 mL of water containing 24 molar equivalents of NaOH (33 µL of a stock solution 1M). Then, 12 mol. eq. (0.84 µmol, 32 mM) of BTA, 12 mol. eq. (0.84 µmol, 74 mM) of THP and 12 mol. eq. (0.84 µmol, 2.38 mM) of IMD were separately added to three aliquots of this COONaRhMOP aqueous solution (0.07 µmol, 0.27 mM). Such addition induced a color change from blue to purple for BTA and IMD, whereas the color remained blue when THP was added. UV-Vis spectra were recorded both from the initial and the final aqueous solutions.

S2. Characterization

S2.1 Model amide coupling between COOHRhMOP and propylamine

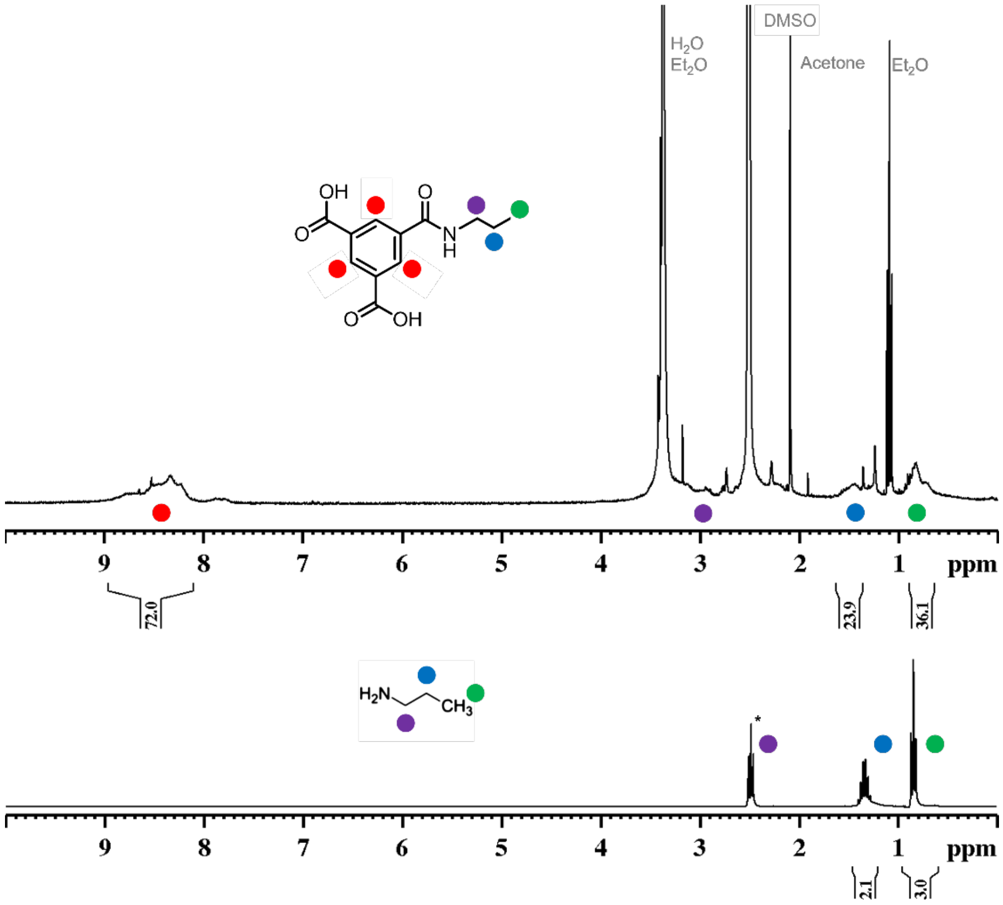

Figure S3. ^{1}H-NMR spectra in DMSO-d_6 of the obtained product from the model amide coupling using COOHRhMOP and propylamine. Note that the aliphatic signals ascribed to the model amine compound became broader upon the amide coupling reaction due to the high molecular weight of the obtained product (*vide infra*). Furthermore, the aliphatic protons closer to the amine of the propylamine are downfield shifted due to the formation of the amide coupling. Star tags the residual DMSO signal.

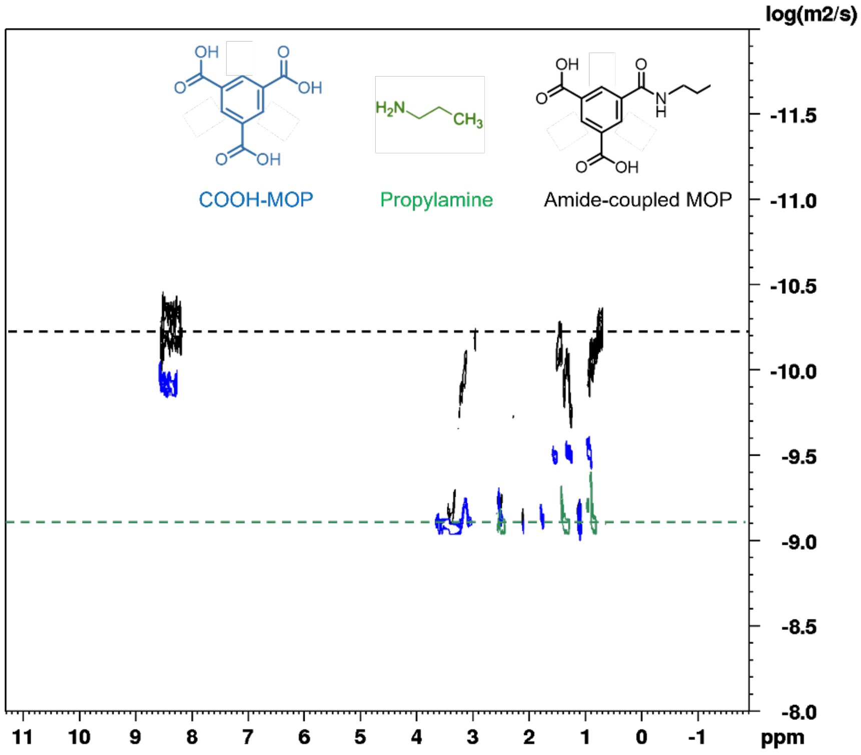

Figure S4. Overlapped DOSY spectra in DMSO-d_6 of the obtained product from the model amide coupling using COOHRhMOP and propylamine (black spectrum) and both of the starting reagents, COOHRhMOP (blue spectrum) and propylamine (green spectrum). The same diffusion coefficient (D = $6.3\cdot10^{-11}$ $m^2\cdot s^{-1}$) is found for the aromatic and the aliphatic signals attributed to the amide coupling product, thus confirming that they all belong to the same molecular entity. Note that, the diffusion coefficient found after the reaction (D = $6.3\cdot10^{-11}$ $m^2\cdot s^{-1}$) decreases with respect to the corresponding value for the free molecule (D = $7.9\cdot10^{-10}$ $m^2\cdot s^{-1}$), demonstrating that the aliphatic protons belong to a larger molecular entity after the amide coupling reaction.

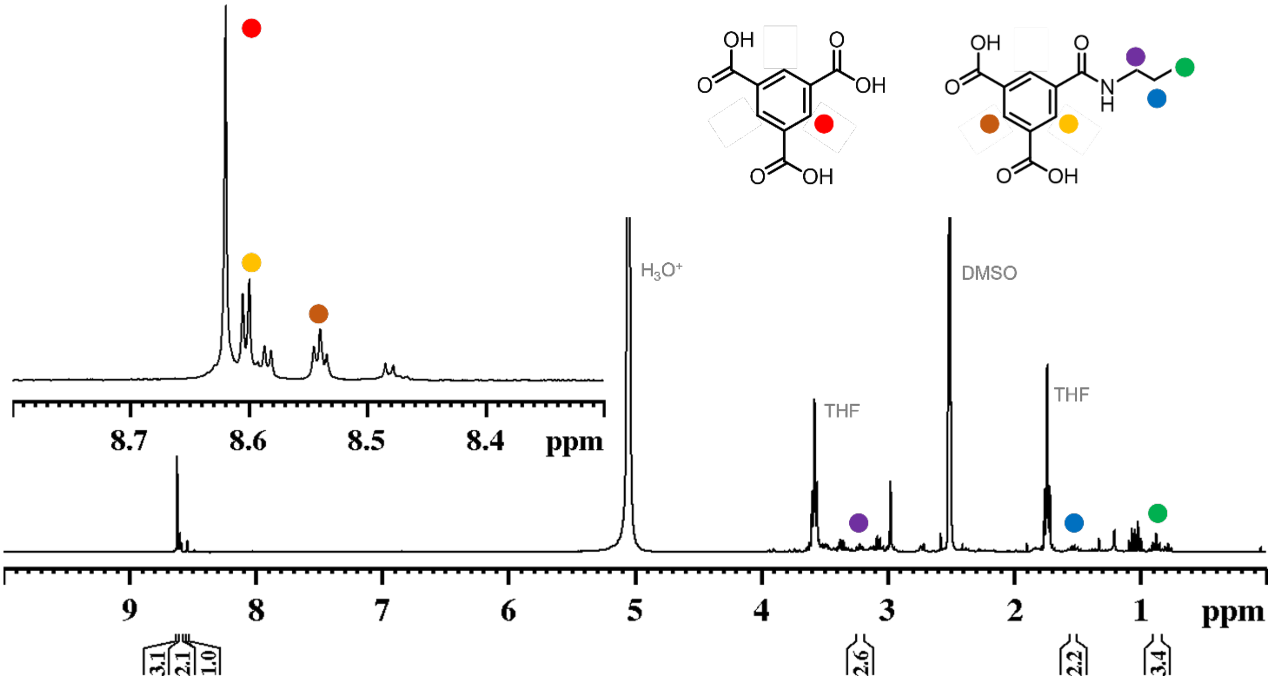

Figure S5. ^{1}H-NMR spectrum in DMSO-d_6 of the digested product obtained from the model amide coupling reaction. Digestion reaction was carried out in strong acidic conditions (750 µl of DMSO-d6 and 20 ul of concentrated DCl (35 wt. %)) at 100ºC for 2 hours. The partial conversion of surface the surface COOH groups of the MOP generates two types of ligands on the surface of the post-functionalized MOP. These ligands are: 1,3,5-benzentricarboxylate (btc) and 5-(((propylamino)oxy)carbonyl)isophthalic acid. The digestion of the post-functionalized MOP liberates these two types of molecules. Thus, the ratio between btc and 5-(((propylamino)oxy)carbonyl)isophthalic acid allowed us to determine the yield of the amide coupling om the surface of the MOP. The relative integration of the aromatic protons of btc (red dot) and 5-(((propylamino)oxy)carbonyl)isophthalic acid (yellow and brown dots) revealed an equimolar ratio between btc and 5-(((propylamino)oxy)carbonyl)isophthalic acid. Therefore, the yield of the coupling between COOH-RhMOP and propylamine was ca. 50%.

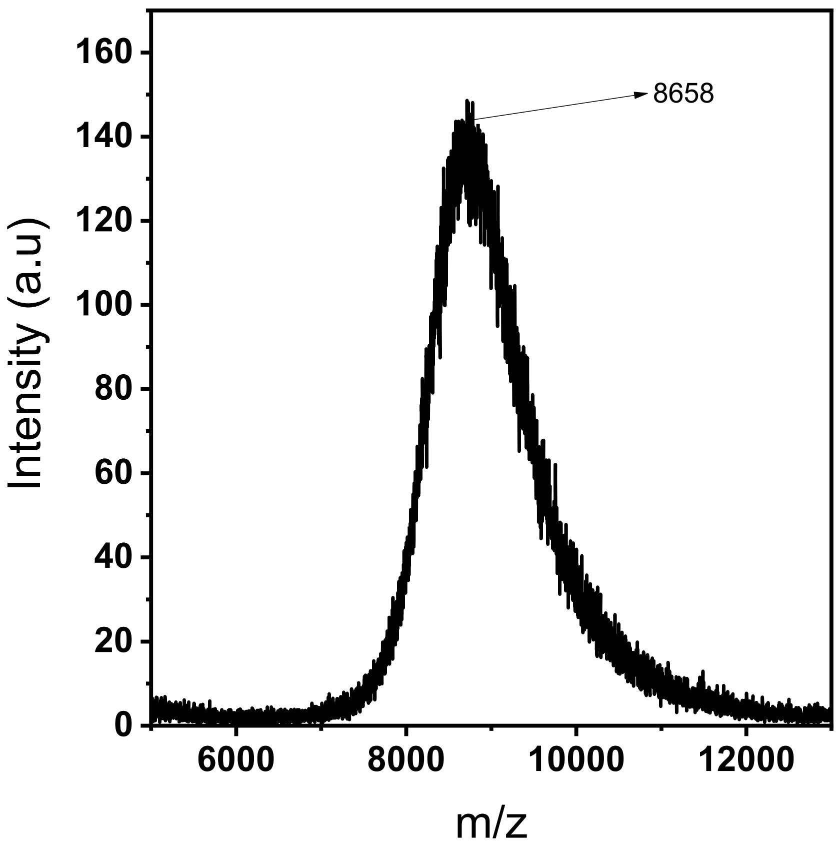

Figure S6. MALDI-TOF spectrum of the obtained product from the model amide coupling reaction. The peak corresponding $[Rh_{24}(COOH\text{-}bdc)_{12}(Amide\text{-}bdc)_{12}(DMSO)_9H^+]^+$ is highlighted (expected = 8661, found = 8658).

S2.2 Coordination capabilities of COOHRhMOP in aqueous solutions

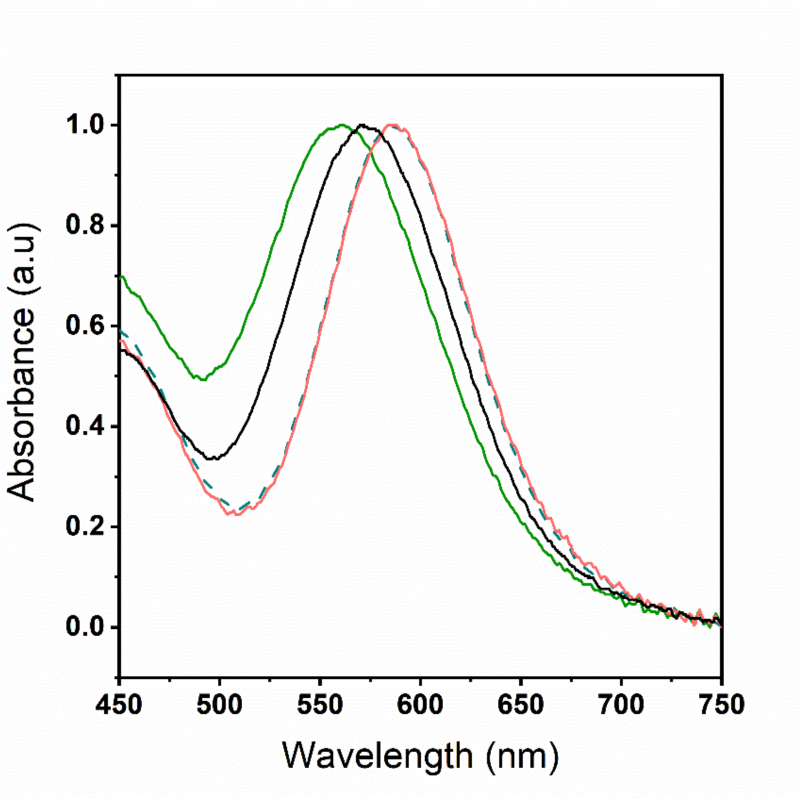

Figure S7. UV-Vis spectra of COONaRhMOP in water before (blue dash line) and after the addition of 12 mol. eq. of BTA (green line), IMD (black line) or THP (pink line). The largest shift of λ_{max} of Band I upon addition of BTA with respect to IMD is attributed to the higher coordination capabilities of BTA *vs* IMD. Upon the addition of THP, the λ_{max} of Band I remained constant confirming the absence of coordinative interaction between this molecule and the Rh(II) open metal sites of the MOP.

S2.3 BiMW sensor surface characterization

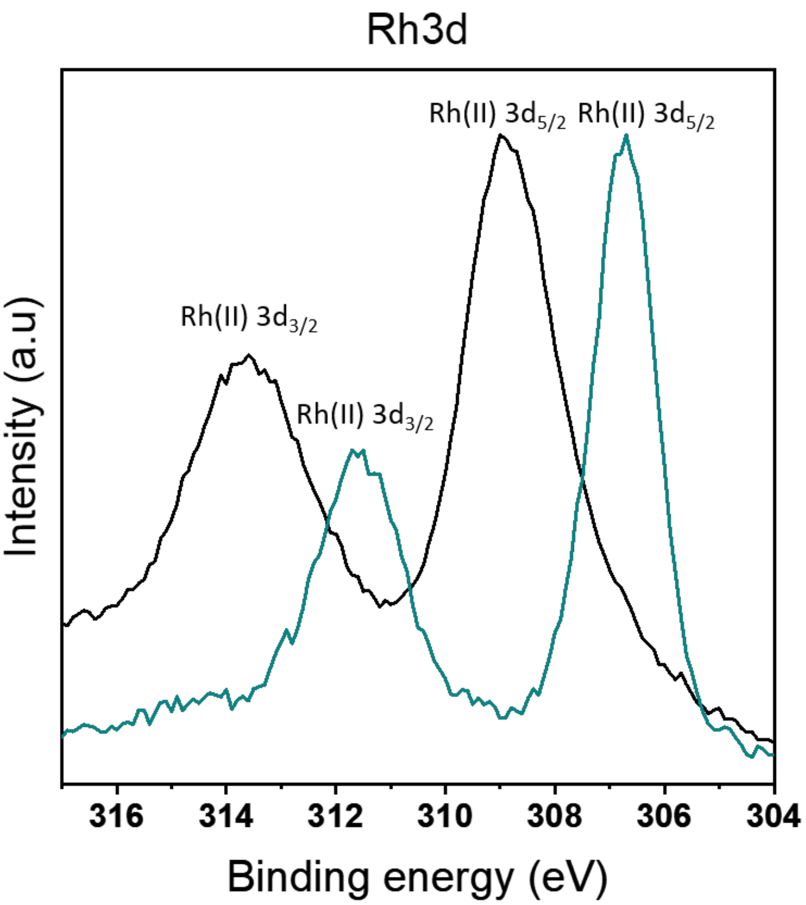

Figure S8. XPS analysis showing the presence of the metal Rh on the BiMW sensor surface with immobilized COOHRhMOP by covalent bonding (black) and the bulk COOHRhMOP in solid state (blue). The values of *ca.* 313 and 311 eV for the Rh(II) $3d_{3/2}$ and of *ca.* 309 and 306 eV for the Rh(II) $3d_{5/2}$ confirm the presence of Rh(II) in both samples.[6a] The slight shifting on the binding energy values can be attributed to the amide bond used to covalently anchor the COOHRhMOP to the chip surface.[7]

Table S1. Rh content through ICP-MS analysis of digested BiMW chips for blank tests and after the covalent immobilization of the $Rh_2(bdc)_4$ cluster and COOHRhMOP.

Condition	Rh (mg/kg)	
	**Mean**	**SD**
**Bare chip (n=1)**	<0,01	0
**APTES (n=2)**	<0,01	0
**$Rh_2(bdc)_4$ (n=2)**	0,76	0,051
**COOHRhMOP (n=3)**	1,30	0,008

S2.4 Characterization of the analytical performance of the MOP-BiMW sensor surface.

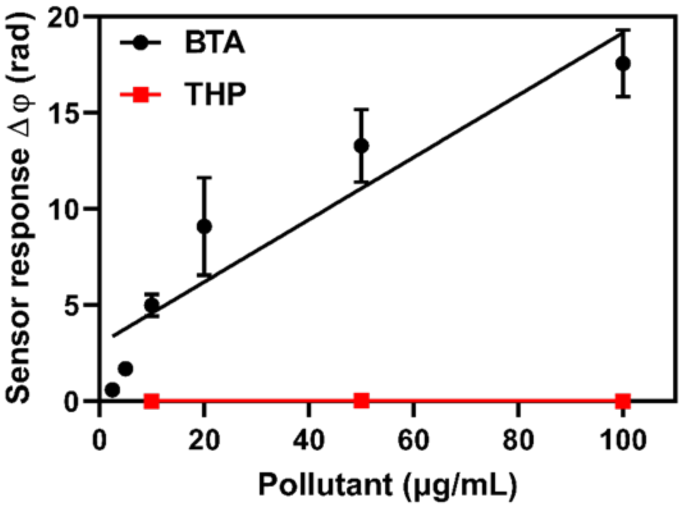

Figure S9. Calibration curves for BTA and THP in the low concentration range (0 – 100 µg/mL). In both calibration curves, each signal corresponds to the mean ± SD of triplicate measurements on different batches of BiMW sensor chips.

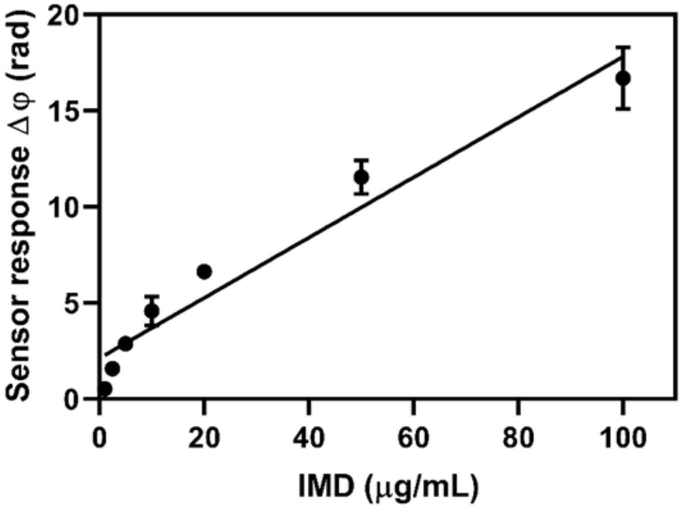

Figure S10. Calibration curves for IMD in the low concentration range (0 – 100 µg/mL). In the calibration curve, each signal corresponds to the mean ± SD of triplicate measurements on different batches of BiMW sensor chips.

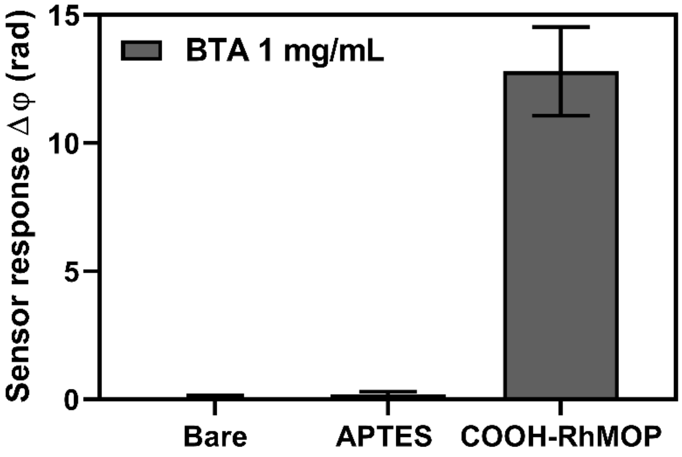

Figure S11. The response of sensors with different surface functionalization to a solution of BTA at a concentration of 1mg/ml.

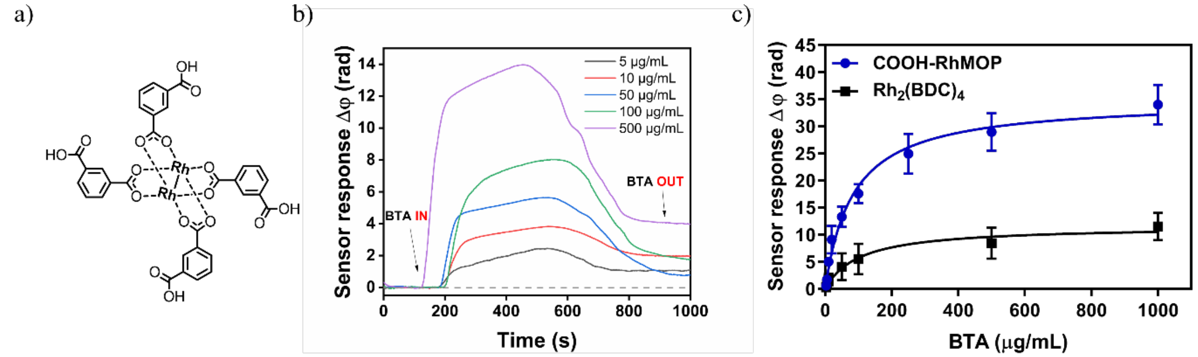

Figure S12. a) Representation of the $Rh_2(BDC)_4$ cluster b) Real-time sensorgram of different concentrations of BTA with the receptor $Rh_2(bdc)_4$ in Milli-Q water. c) Calibration curves for BTA in Milli-Q water using $Rh_2(bdc)_4$ (black line) and COOHRhMOP (blue line) as chemical receptors. In both calibration curves each signal corresponds to the mean ± SD of triplicate measurements.

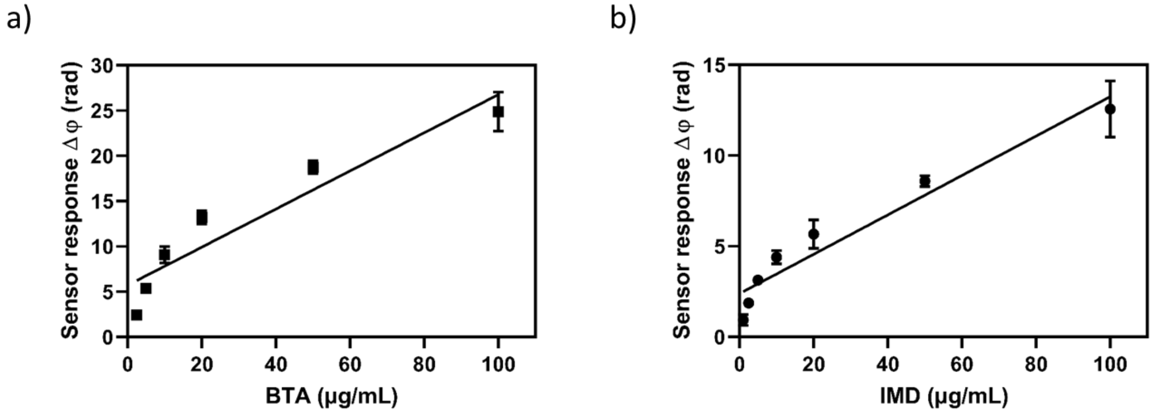

Figure S13. Calibration curves for BTA (a) and IMD (b) in the low concentration range (0 – 100 µg/mL). In the calibration curves, each signal corresponds to the mean ± SD of triplicate measurements on different batches of BiMW sensor chips.

Table S2 Analytical and performance comparison between the MOP-based sensor and existing procedures.

**Technique**	**Pre-treatment**	**Linear range (µg/mL)**	**LoD (µg/mL)**	**Matrix**	**Tap water samples**	**Reference**
			BTA			
**HPLC**	Liquid-Liquid Extraction	2-100	2.04	Transformer oil	No	***8***
**SERS**	Colloidal Lignin Particles	1-300	0.12	Transformer oil	No	***9***
**SERS**	Wastewater samples were concentrated 2 or 4 times	-	0.018	Milli-Q water	Yes	***10***
**DPV**	None	1.2-8	0.44	Milli-Q water	Yes	***11***
**BiMW-MOP**	None	2.5-100	0.064	Milli-Q water	Yes	***This work***
			**IMD**			
**HPLC**	Solid-Phase Extraction	0.01 -10	0.0005	Soil and Milli-Q water	No	***12***
**MIPs**	None	1.3-25.6	1.18	Milli-Q water	Yes	***13***
**MCS**	None	0-255	0.65	Milli-Q water	Yes	***14***
**BiMW-MOP**	None	1-100	0.234	Milli-Q water	Yes	***This work***

HPLC: High-Performance Liquid Chromatography
SERS: Surface-Enhance Raman Scattering
DPV: Differential Pulse Voltammetry
BiMW-MOP: Bimodal Waveguide platform with Metal Organic Polyhedra
MIP: Molecularly Imprinted Polymers
MCS: Microporous Carbon Sensor

3. Data analysis

Data were analyzed using Origin 8.0 (OriginLab) and GraphPad Prism (Graphpad Software, US). Calibration curves were plotted as mean ± SD (standard deviation) of the accumulated biosensor response signals in triplicate (after signal stabilization at t = 1000 s) versus the accumulated BTA/IMD concentrations. The data were fitted to one-site specific binding model regression according to the following formula (eq.1):

$$y = (A \cdot X)/((B+X)) \quad \text{(Eq. 1)}$$

where y is the sensor response, X is the concentration of BTA/IMD, A is the extrapolated maximum number of receptor sides on the surface and B is the equilibrium binding constant which corresponds to the analyte concentration needed to achieve half-maximum receptor sides occupied at equilibrium.

The limit of detection (LOD) is defined as the minimum variation of analyte concentration that produces a signal at least three times higher than the detectable noise of the system which corresponds to the standard deviation (SD) of the baseline in between measurements. Therefore, LoD was calculated as the concentration corresponding to the blank signal plus three times its SD.

The coefficient of variation (CV) between measurements is evaluated according to equation 2, where μ is the mean and σ the SD:

$$CV(\%) = \sigma/\mu \times 100 \qquad \text{(Eq. 2)}$$

To evaluate the accuracy of the assay, spiked samples in tap water were prepared by spiking known concentrations of BTA/IMD. The concentrations evaluated by the sensing platform were calculated by interpolating the signal into the calibration curve. Accuracy was determined by applying equation 3:

$$\text{Accuracy}(\%) = [\text{analyte}]\text{calculated}/[\text{analyte}]\text{real} * 100 \qquad \text{(Eq. 3)}$$

4. References

(1) Albalad, J.; Carné-Sánchez, A.; Grancha, T.; Hernández-López, L.; Maspoch, D. Protection Strategies for Directionally-Controlled Synthesis of Previously Inaccessible Metal–Organic Polyhedra (MOPs): The Cases of Carboxylate-and Amino. *Chem. Commun.* **2019**, *55*, 12785-12788.

(2) Baeckmann, C. von; Ruiz-Relaño, S.; Imaz, I.; Handke, M.; Juanhuix, J.; Gándara, F.; Carné-Sánchez, A.; Maspoch, D. Stepwise Assembly of Heterometallic, Heteroleptic "Triblock Janus-Type" Metal–Organic Polyhedra. *Chem. Commun.* **2023**, 59, 3423-3426.

(3) Zinoviev, K.; González-Guerrero, A. B.; Domínguez, C.; Lechuga, L. M. Integrated Bimodal Waveguide Interferometric Biosensor for Label-Free Analysis. *J. Lightwave Technol.* **2011**, *29* , 1926-1930.

(4) Bassols-Cornudella, B.; Ramirez-Priego, P.; Soler, M.; Estévez, M.-C.; Díaz Luis-Ravelo, H. J.; Cardenosa-Rubio, M.; Lechuga, L. M. Novel Sensing Algorithm for Linear Read-Out of Bimodal Waveguide Interferometric Biosensors. *J. Lightwave Technol.* **2022**, *40*, 237-244.

(5) Calvo-Lozano, O.; García-Aparicio, P.; Raduly, L. Z.; Estévez, M. C.; Berindan-Neagoe, I.; Ferracin, M.; Lechuga, L. M. One-Step and Real-Time Detection of MicroRNA-21 in Human Samples for Lung Cancer Biosensing Diagnosis. *Anal. Chem.* **2022**, *94*, 14659–14665.

(6) Shi, Y.; Hu, X.; Zhu, B.; Wang, S.; Zhang, S.; Huang, W. Synthesis and Characterization of TiO_2 Nanotube Supported Rh-Nanoparticle Catalysts for Regioselective Hydroformylation of Vinyl Acetate. *RSC Adv.* **2014**, *4*, 62215-62222.

(7) Ghosh, A. C.; Legrand, A.; Rajapaksha, R.; Craig, G. A.; Sassoye, C.; Balázs, G.; Farrusseng, D.; Furukawa, S.; Canivet, Florian J.; Wisser, M. W,; Rhodium-Based Metal-Organic Polyhedra Assemblies for Selective CO_2 Photoreduction. *J. Am. Chem. Soc.* **2022**, *144*, 3626-3636.

(8) RefA: Jaber, A. M. Y.; Mehanna, N. A.; Abulkibash, A. M. Simultaneous Liquid–Liquid Extraction of Dibenzyl Disulfide, 2, 6-di-tert-butyl-p-cresol, and 1, 2, 3-Benzotriazole from Power Transformer Oil prior to GC and HPLC Determination. *J. Sep. Sci.* **2012**, *35*, 750-757.

(9) Shen, Y.; Yu, D.; Han, F. Y.; Shen, A. G.; Hu, J. M. On-Site and Quantitative SERS Detection of Trace 1, 2, 3-Benzotriazole in Transformer Oil with Colloidal Lignin Particles-Based Green Pretreatment Reagents. *Spectrochim. Acta A Mol. Biomol. Spectrosc.* **2021**, *252*, 119469.

(10) RefB: Wieduwilt, F.; Lenth, C.; Ctistis, G.; Plachetka, U.; Möller, M.; Wackerbarth, H. Evaluation of an On-Site Surface Enhanced Raman Scattering Sensor for Benzotriazole. *Sci. Rep.*, **2020**, *10*, 8260.

(11) Muschietti, A.; Serrano, N.; Ariño, C.; Díaz-Cruz, M. S.; Díaz-Cruz, J. M. Screen-Printed Electrodes for the Voltammetric Sensing of Benzotriazoles in Water. *Sensors* **2020**, *20*, 1839.

(12) Baskaran, S.; Kookana, R. S.; Naidu, R. Determination of the Insecticide Imidacloprid in Water and Soil Using High-Performance Liquid Chromatography. *J. Chromatogr. A* **1997**, *787*, 271-275.

(13) El-Akaad, S.; Mohamed, M. A.; Abdelwahab, N. S.; Abdelaleem, E. A.; De Saeger, S.; Beloglazova, N. Capacitive Sensor Based on Molecularly Imprinted Polymers for Detection of the Insecticide Imidacloprid in Water. *Sci. Rep.* **2020**, *10*, 1–10.

(14) Kaewket, K.; Ngamchuea, K. Microporous Carbon for Fast and Simple Electrochemical Detection of Imidacloprid Insecticide in Fruit and Water Samples. *RSC Adv.* **2023**, *13*, 4532-4541.
